# Pasteurized *Akkermansia muciniphila* Ameliorates Preeclampsia in Mice by Enhancing Gut Barrier Integrity, Improving Endothelial Function, and Modulating Gut Metabolic Dysregulation

**DOI:** 10.3390/microorganisms12122483

**Published:** 2024-12-02

**Authors:** Linyu Peng, Qinlan Yin, Xinwen Wang, Yawen Zhong, Yu Wang, Wanting Cai, Ruisi Zhou, Ying Chen, Yu Hu, Zhixing Cheng, Wenqian Jiang, Xiaojing Yue, Liping Huang

**Affiliations:** Department of Obstetrics and Gynecology, Nanfang Hospital, Southern Medical University, Guangzhou 510515, China; 13253666550@163.com (L.P.);

**Keywords:** preeclampsia, *Akkermansia muciniphila*, pasteurization, endothelial function, metabolomics

## Abstract

Preeclampsia (PE) is a serious complication of pregnancy linked to endothelial dysfunction and an imbalance in the gut microbiota. While *Akkermansia muciniphila* (AKK) has shown promise in alleviating PE symptoms, the use of live bacteria raises safety concerns. This study explored the potential of pasteurized *A. muciniphila* (pAKK) as a safer alternative for treating PE, focusing on its effects on endothelial function and metabolic regulation. A PE mouse model was induced via the nitric oxide synthase inhibitor L-NAME, followed by treatment with either pAKK or live AKK. Fecal metabolomic profiling was performed via liquid chromatography–tandem mass spectrometry (LC-MS/MS), and in vivo and in vitro experiments were used to assess the effects of pAKK on endothelial function and metabolic pathways. pAKK exhibited therapeutic effects comparable to those of live AKK in improving L-NAME-induced PE-like phenotypes in mice, including enhanced gut barrier function and reduced endotoxemia. pAKK also promoted placental angiogenesis by restoring endothelial nitric oxide synthase (eNOS) activity and nitric oxide (NO) production. The in vitro experiments further confirmed that pAKK alleviated L-NAME-induced NO reduction and endothelial dysfunction in human umbilical vein endothelial cells (HUVECs). Metabolomic analysis revealed that both pAKK and live AKK reversed metabolic disturbances in PE by modulating key metabolites and pathways related to unsaturated fatty acid biosynthesis, folate, and linoleic acid metabolism. As a postbiotic, pAKK may support existing treatments for preeclampsia by improving gut barrier function, restoring endothelial function, and regulating metabolic dysregulation, offering a safer alternative to live bacteria. These findings highlight the potential clinical value of pAKK as an adjunctive therapy in managing PE.

## 1. Introduction

Preeclampsia (PE) is a complex pregnancy-related disorder with a global incidence of 2–8% [1], and it is one of the leading causes of maternal and neonatal morbidity and mortality [2]. Clinically, PE is characterized by hypertension and proteinuria. Its pathogenesis involves multiple factors, including placental factors, immune imbalance, genetic susceptibility, and metabolic disturbances [3]. Vascular endothelial dysfunction is a key feature, with hypoxia and placental inflammatory mediators leading to vasospasm and hypertension [4,5]. Currently, PE treatment focuses on blood pressure control and symptom management, with pregnancy termination being the most effective measure to prevent severe complications [3]. Given the complex pathophysiology of PE, developing new therapeutic strategies is crucial.

Recent studies have increasingly highlighted the role of the gut microbiota in the pathogenesis of PE. Emerging evidence suggests that gut microbiota dysbiosis exacerbates systemic inflammation and endothelial dysfunction, thereby accelerating PE progression [6,7]. Among the gut commensals, *Akkermansia muciniphila* (AKK) has shown considerable promise in alleviating metabolic disorders such as obesity and diabetes by enhancing gut barrier function, regulating microbial composition, and maintaining metabolic homeostasis [8]. In PE mouse models, AKK has been reported to modulate immune responses and promote spiral artery remodeling through its metabolites, such as short-chain fatty acids (SCFAs), thereby mitigating PE symptoms [9]. Moreover, AKK has been suggested to improve hypertension by regulating the nitric oxide (NO) pathway [10,11], a mechanism that plays an important role in PE treatment.

However, the use of live bacteria presents safety concerns, such as the risk of dysbiosis or infection. As a safer alternative, pasteurized *A. muciniphila* (pAKK) has been proposed. Studies have demonstrated that pAKK is equally, if not more effective than live bacteria, in treating obesity and metabolic syndrome, with significantly reduced safety risks due to pasteurization [12]. In 2021, pAKK was approved by the European Food Safety Authority (EFSA) as a novel food [13], further highlighting its potential for both food and medical applications. pAKK has been shown to improve gut barrier integrity by modulating Toll-like receptor 2 (TLR2), reducing lipopolysaccharide (LPS)-induced intestinal permeability and systemic inflammation [14]. Additionally, pAKK exerts immunoregulatory effects by reducing TNF-α and increasing IL-10 levels in macrophages [15]. In models of liver fibrosis induced by a high-fat diet and CCl4, pAKK inhibited hepatic stellate cell activation, reduced fibrosis and inflammatory markers, and improved gut permeability [16]. These findings underscore the broad therapeutic potential of pAKK in metabolic regulation, liver diseases, and gut health.

Despite these promising findings in metabolic disorders, the role of pAKK in PE has not been thoroughly investigated. This study aims to systematically assess the therapeutic potential of pAKK in PE, with a particular focus on its regulatory effects on endothelial function. Using an N(G)-nitro-L-arginine methyl ester (L-NAME)-induced PE mouse model, we evaluated the impact of pAKK on endothelial function, placental angiogenesis, and gut barrier function. Additionally, fecal metabolomics analysis was conducted to explore the underlying metabolic regulatory mechanisms. This research provides novel insights into microbiota-targeted therapies for PE and offers evidence to support the development of safe and effective clinical interventions for this condition.

## 2. Methods

### 2.1. Culturing of AKK and Preparation of pAKK

The AKK strain (ATCC BAA-835) was cultured in thioglycolate medium at 37 °C under strictly anaerobic conditions (80% N_2_, 10% H_2_, and 10% CO_2_) and stored at −80 °C until further use. pAKK was prepared as previously described [8]. Briefly, the grown AKK was centrifuged at 12,000× *g* for 10 min to remove the medium, rinsed twice, and then suspended in 0.01 M PBS, followed by pasteurization at 70 °C for 30 min. Before use, AKK and pAKK were diluted with sterile PBS containing 2.5% glycerol to a concentration of 1 × 10^8^ CFU/mL.

### 2.2. Animals and Experimental Protocol

The animal study was conducted in accordance with the Guide for the Care and Use of Laboratory Animals by the National Institutes of Health (NIH) and was approved by the local Animal Care and Use Committee of Southern Medical University (approval code: L2020098; approval date: 11 September 2020). All procedures were performed under sodium pentobarbital anesthesia to minimize animal discomfort.

C57BL/6J mice (6–8 weeks old) were purchased from the Experimental Animal Center of Southern Medical University. The mice were housed in a controlled environment (50 ± 10% relative humidity, 12/12 h light/dark cycle, 22 ± 2 °C) with free access to standard food and water. The female mice received 200 µL of AKK, pAKK (1 × 10^8^ CFU/mL), or an equal volume of vehicle (sterile PBS containing 2.5% glycerol) via oral gavage every other day starting from two weeks prior to pregnancy confirmation and continuing until the end of the experiment. They were then mated overnight with male mice at a 2:1 ratio, and the day of confirmed pregnancy, indicated by the presence of a vaginal plug, was designated as E0.5. Following overnight mating, six pregnant mice from each group were randomly selected via a random number generator, and the remaining mice were excluded. Animals with health issues such as significant weight loss or unexpected illnesses were excluded from the study, and their exclusion was documented. To model PE, the pregnant mice were administered L-NAME (125 mg/kg/day, NG-nitro-L-arginine methyl ester hydrochloride, HY-18729A, MedChemExpress, Monmouth Junction, NJ, USA) via oral gavage daily from E8.5 for 10 days. The mice were subdivided into two groups, those receiving either L-NAME (PLN group) or the vehicle control (CTRL group). Other mice that received AKK (AmLN group) or pAKK (pAmLN group) were also gavaged with L-NAME. No adverse events were observed during the study. The animals were monitored daily for health and welfare.

Systolic blood pressure (SBP) was measured via a noninvasive tail-cuff blood pressure instrument (Softron Biotechnology, Beijing, China) on the day of initiation and on E0.5, E4.5, E8.5, E12.5, E16.5, and E18.5, between 9 and 11 AM. Urine samples were collected on E18.5 using metabolic cages over a 6 h period. After collection, the urine was centrifuged at 800× *g* for 5 min at 4 °C to remove debris and stored at −80 °C for subsequent albumin analysis. Fecal samples were freshly collected using sterile forceps, snap-frozen in liquid nitrogen, and stored at −80 °C until analysis. Untargeted metabolomics analysis of the fecal samples was performed via liquid chromatography–tandem mass spectrometry (LC-MS/MS) [17]. On E18.5, the mice were anesthetized with 1.5% pentobarbital (60 mg/kg) to collect blood serum, which was subsequently centrifuged at 3000× *g* at 4 °C for 15 min to separate the serum. The intestines and uterus were then harvested, and the small intestine, colon, fetuses, and placentas were dissected, measured, and weighed. The live birth rate, fetal weight, crown-rump length, and fetal/placental weight ratio were recorded. Organs were either flash-frozen and stored at −80 °C for further analysis or immediately fixed in 4% (*v*/*v*) paraformaldehyde or Carnoy’s fixative, followed by standard paraffin-embedding procedures.

### 2.3. Hematoxylin and Eosin (HE) Staining and Immunofluorescence (IF) of the Placenta

For histological evaluation, placentas were fixed in 4% neutral-buffered paraformaldehyde for 12–16 h at 20–25 °C. The tissues were then infiltrated, embedded in paraffin, and sectioned into 4 μm slices. Hematoxylin and eosin (HE) staining was performed via standard techniques on these paraffin sections for a conventional morphological evaluation with a NanoZoomer S360 digital slide scanner (Hamamatsu Photonics, Hamamatsu, Shizuoka, Japan). The structure of the placenta implantation site was observed, the ratio between the two functional areas of the placenta (labyrinth and junctional zone) was calculated, and the infarct size of the placenta was assessed. To evaluate angiogenesis in the placenta, a regular immunofluorescence (IF) assay was performed. Briefly, 4 μm paraffin sections were cut, deparaffinized, and subjected to antigen retrieval. The sections were then incubated overnight at 4 °C with a CD31 antibody (1:1000; ab182981, Abcam, Cambridge, UK). Fluorescence images were captured via a Pannoramic MIDI scanner (3D HISTECH, Budapest, Hungary), and the data were analyzed via CaseViewer software (version 2.4) to quantify the CD31-positive areas and vessel density.

### 2.4. Intestinal Barrier Function Assessment

Distal colon tissues were collected and fixed in 4% paraformaldehyde at room temperature for 24 h. The tissues were then embedded in paraffin and sectioned into 4 µm slices. HE and alcian blue periodic acid Schiff (AB-PAS) staining were performed on the paraffin sections of each colon, following standard protocols. For each mouse, 17 to 23 crypts (3–5 crypts per colonic section) were randomly selected to quantify the number of goblet cells per crypt and the crypt depth and to conduct histological injury scoring. These quantitative analyses were performed via ImageJ software (v.1.53t) with the appropriate cell-counting plugins. For occludin immunohistochemistry (IHC) staining, paraffin-embedded sections were deparaffinized in xylene, rehydrated through graded ethanol, and subjected to antigen retrieval in sodium citrate buffer (10 mM, pH 6.0) at 95 °C for 15 min. Sections were blocked with 3% hydrogen peroxide for 10 min to quench endogenous peroxidase activity, followed by blocking with 5% bovine serum albumin for 30 min at room temperature. The sections were then incubated overnight at 4 °C with a primary antibody against occludin (1:200, TD7504, Abmart, Shanghai, China). After washing in phosphate-buffered saline (PBS), the sections were incubated with an HRP-conjugated secondary antibody for 30 min at room temperature. Signal development was performed using a DAB substrate kit (Servicebio, Wuhan, China), and the nuclei were counterstained with hematoxylin. The stained sections were visualized under a microscope, and the intensity of occludin staining was quantified using ImageJ software. Additionally, the plasma LPS levels were measured via a competitive ELISA kit (Meimian, Yancheng, China) following the manufacturer’s instructions.

### 2.5. Enzyme-Linked Immunosorbent Assay (ELISA)

Serum soluble Fms-like tyrosine kinase-1 (sFlt-1), placental growth factor (PlGF), and tetrahydrobiopterin (BH4) levels were quantitatively analyzed via commercial sandwich ELISA kits (Meimian, Yancheng, China) according to the manufacturer’s instructions. The urine samples were centrifuged at 800× *g* for 5 min at 4 °C to remove debris before analysis. The albumin concentration in the urine was determined via a mouse ALB ELISA kit (Bioswamp, Wuhan, China) following the manufacturer’s protocol. The optical density was measured at 450 nm via a microplate reader (Bio-Rad, Hercules, CA, USA). The concentrations of serum sFlt-1, PlGF, BH4, and urinary albumin were calculated on the basis of the standard curves generated with known concentrations of the standards provided in the kits.

### 2.6. Determination of Serum Nitric Oxide (NO)

The serum nitric oxide (NO) levels were determined via the Total Nitric Oxide Assay Kit (Beyotime, Shanghai, China) according to the manufacturer’s instructions. The serum samples were stored at −20 °C for no longer than two weeks prior to analysis. The assay procedure involved diluting the samples or standards to the desired concentrations, followed by the sequential addition of 2 mM NADPH (Nicotinamide adenine dinucleotide phosphate) solution, FAD (flavin adenine dinucleotide) solution, nitrate reductase, LDH (lactate dehydrogenase) buffer, and LDH enzyme solution. After incubation at 37 °C, Griess Reagent I and Griess Reagent II were added, and the mixture was incubated at room temperature in the dark for 10 min. The absorbance was measured at 540 nm via a microplate reader (Bio-Rad, Hercules, CA, USA), and the NO concentrations in the samples were calculated via a standard curve.

### 2.7. Cell Culture

Human umbilical vein endothelial cells (HUVECs) were purchased from IMMOCELL (Xiamen, China). The cells were cultured in RPMI 1640 medium (Pricella Life Science & Technology Co., Ltd., Wuhan, China) supplemented with 10% fetal bovine serum (FBS) and 1% penicillin-streptomycin (both from Procell, Wuhan, China). HUVECs between passages 3 and 7 were used for all the experiments to maintain consistency and functionality. The cells were maintained at 37 °C in a humidified incubator with 5% CO_2_. Once the cells reached approximately 70% confluence, they were either passaged or used for subsequent experiments.

### 2.8. Measurement of Intracellular NO Levels

The intracellular NO levels were measured via the fluorescent probe DAF-FM DA (4-Amino-5-Methylamino-2′,7′-Difluorofluorescein Diacetate, Beyotime Biotechnology, Shanghai, China). HUVECs were seeded in 6-well plates and cultured under standard conditions (37 °C, 5% CO_2_, 95% humidity) until 70–80% confluence. The cells were treated with 300 μM L-NAME and 1 × 10^7^ CFU/mL pAKK for 24 h. After treatment, the cells were incubated with 5 µM DAF-FM DA in a serum-free medium at 37 °C for 20 min in the dark. Excess dye was removed by washing three times with phosphate-buffered saline (PBS, pH 7.4). For qualitative analysis, fluorescence was visualized using a fluorescence microscope (excitation 495 nm, emission 515 nm). For quantitative analysis, the cells were harvested, resuspended in PBS, and analyzed by flow cytometry (BECKMAN CytoFlex S, Beckman Coulter, Brea, CA, USA) at an excitation of 488 nm and emission of 530 nm. Data were processed with CytExpert software (v.2.1).

### 2.9. HUVEC Angiogenesis Assay

For the angiogenesis assay, 50 µL of Matrigel matrix (Beyotime, Shanghai, China) was added to each well of a precooled 96-well plate to prevent premature solidification. The plate was then incubated at 37 °C in a CO_2_ incubator for 30 min to allow the Matrigel to solidify completely. HUVECs were suspended in the appropriate medium at a concentration of 3 × 10^5^ cells/mL, and 50 µL of the cell suspension (1.5 × 10^4^ cells/well) was seeded onto the solidified Matrigel in each well. The plate was incubated at 37 °C in a humidified CO_2_ incubator for 5 h to facilitate tube formation. Tube formation was observed and imaged via an inverted microscope. Quantitative analysis of the total tube length, number of meshes, number of junctions, and percentage of mesh area was performed via ImageJ software with the Angiogenesis Analyzer plugin.

### 2.10. RNA Isolation and Quantitative Real-Time PCR (qRT-PCR)

Total RNA was isolated from the placenta, jejunum, ileum, colon, and HUVECs via the TRIzol Reagent (Thermo Fisher Scientific, Waltham, MA, USA) following the manufacturer’s protocol. The RNA concentration and purity were determined via a NanoDrop ultramicro UV spectrophotometer (Thermo Scientific, USA), with the A260/A280 ratios between 1.8 and 2.0 considered indicative of high purity. The RNA was reverse transcribed into cDNA via a high-capacity cDNA reverse transcription kit (Vazyme, Nanjing, China). Real-time PCR was performed with the SYBR Green PCR Master Mix on a 7500 FAST Real-Time PCR System (Applied Biosystems, Foster City, CA, USA). Human and mouse gene primers were synthesized by OriGene Technologies, Inc. (Rockville, MD, USA), and their sequences are listed in Supplementary Table S1. The gene expression levels were calculated via the 2^−ΔΔCt^ method, with GAPDH used as the reference gene for normalization.

### 2.11. Western Blot

Proteins were extracted from cells and placental tissues via the radioimmunoprecipitation assay (RIPA) lysis buffer (Beyotime, Shanghai, China) containing a mixture of protease and phosphatase inhibitors (Beyotime, Shanghai, China). Proteins were separated by sodium dodecyl sulfate-polyacrylamide gel electrophoresis (SDS-PAGE) and transferred to polyvinylidene fluoride (PVDF) membranes. The membranes were blocked with 5% skim milk in TBST for 1 h at room temperature and then incubated overnight at 4 °C with primary antibodies against eNOS (1:1000 dilution, Proteintech, Wuhan, China) and GAPDH (1:2000 dilution, Beyotime, Shanghai, China). After being washed with TBST, the membranes were incubated with HRP-labeled secondary antibodies (1:5000 dilution) for 1 h at room temperature. The bands were visualized via an enhanced chemiluminescence (ECL) substrate (Biosharp, Shanghai, China) and imaged with a Syngene fully automated gel imaging analysis system. The exposure times were optimized to avoid signal saturation. Protein expression was quantified via ImageJ software, with normalization to GAPDH as a loading control, and background correction was performed to ensure accurate quantification.

### 2.12. Metabolomics and Pathway Enrichment Analyses

#### 2.12.1. Sample Collection and Metabolite Extraction

Fecal samples were collected from four groups of mice: CTRL, PLN, AmLN, and pAmLN. Each sample was lyophilized, and the metabolites were extracted by prechilling with 80% methanol (*v*/*v*) following standard protocols. The samples were vortexed, incubated on ice for 5 min, and centrifuged at 15,000× *g* for 20 min at 4 °C. The supernatant was collected, diluted with LC-MS-grade water to 53% methanol, and centrifuged again under the same conditions. The final supernatant was injected into the LC-MS/MS system for analysis.

#### 2.12.2. LC-MS/MS Analysis

Metabolomic profiling was performed via an ultrahigh-performance liquid chromatography (UHPLC) system coupled with a high-resolution Orbitrap Q Exactive™ mass spectrometer (Thermo Fisher Scientific, Waltham, MA, USA). The samples were injected into a Hypersil Gold column (100 × 2.1 mm, 1.9 μm) with a 12 min linear gradient at a flow rate of 0.2 mL/min. The mobile phases were 0.1% formic acid in water (eluent A) and methanol (eluent B). The gradient was set as follows: 2% B for 1.5 min, 2–85% B for 3 min, 85–100% B for 10 min, and 100–2% B for 10.1 min, with re-equilibration at 2% B for 12 min. The mass spectrometer was operated in the positive and negative ion modes with a spray voltage of 3.5 kV, a capillary temperature of 320 °C, a sheath gas flow rate of 35 psi, and an auxiliary gas flow rate of 10 L/min. Data were acquired via the full-scan and data-dependent acquisition modes, covering a scan range of *m*/*z* 100–1000. Mouse fecal metabolomics raw data from the CTRL, PLN, AmLN, and pAmLN groups have been deposited in Mendeley Data and can be accessed at Peng, Linyu (2024) [18].

#### 2.12.3. Data Processing and Statistical Analysis

The raw data were processed via Compound Discoverer 3.3 (Thermo Fisher Scientific, Waltham, MA, USA) for peak alignment, detection, and quantification, with a mass tolerance of 5 ppm and a signal intensity tolerance of 30%. The peak intensities were normalized to the total ion intensity, and the molecular formulas were predicted on the basis of accurate mass, isotopic patterns, and fragment ions. Metabolite identification was performed via the mzCloud, mzVault, and MassList databases. Principal component analysis (PCA) and partial least squares discriminant analysis (PLS-DA) were conducted to visualize the metabolic differences between groups. Differentially abundant metabolites were identified via univariate analysis (*t*-test) with thresholds of VIP > 1, *p* < 0.05, and |log2FC| > 1.5. Volcano plots and Venn diagrams were used to illustrate the differentially abundant metabolites. Pathway enrichment analysis was conducted via the KEGG database (v.94.0), with the statistical significance determined via a hypergeometric test (*p* < 0.05). Visualization of the enriched pathways was performed via dot plots, where the dot size represents the number of metabolites, and the color indicates statistical significance.

### 2.13. Statistical Analysis

All the statistical analyses were performed via GraphPad Prism software (v.8.0). The results are expressed as the means ± SEMs from multiple independent experiments. A Student’s *t*-test was used for comparisons between two groups, whereas the one-way ANOVA followed by Tukey’s post hoc test was employed to analyze the significant differences among multiple groups. In all analyses, a *p* < 0.05 was considered statistically significant. The levels of significance are indicated in the figures as follows: * *p* < 0.05, ** *p* < 0.01, *** *p* < 0.001, **** *p* < 0.0001.

## 3. Results

### 3.1. Pasteurized Akkermansia muciniphila Alleviates the PE Symptoms Induced by L-NAME in Mice

**Figure 1:**
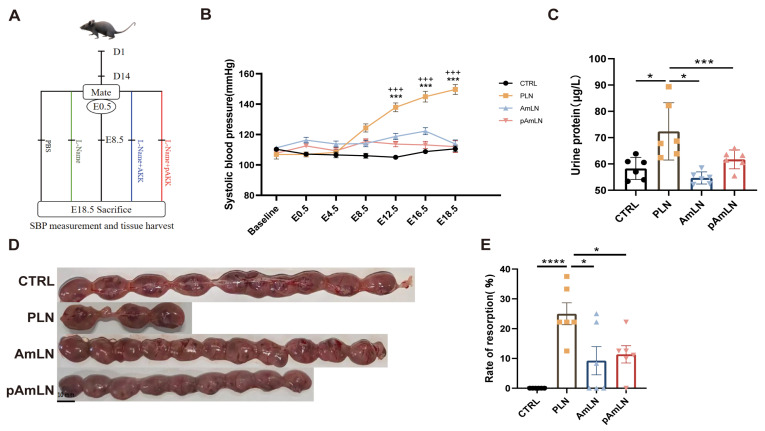
Pasteurized *Akkermansia muciniphila* alleviates hypertension and fetal resorption in PE mouse models. (**A**) Experimental design. To determine whether pasteurized *Akkermansia muciniphila* (pAKK) has the same beneficial effects as live *A. muciniphila* (AKK) in alleviating PE symptoms, the mice were pretreated with PBS, live AKK, or PAKK. At E8.5, the mice were divided into CTRL, PLN, AmLN, and pAmLN groups (n = 6/group) depending on whether they received L-NAME by gavage. (**B**) Systolic blood pressure (SBP). (**C**) Urinary albumin. (**D**) Gross morphology of dissected uteruses showing fetal resorption sites across groups. (**E**) Fetal absorption rate per litter. Data are presented as the mean ± SEM. Significant differences based on one-way ANOVA followed by Bonferroni’s post hoc test: * *p* < 0.05, *** *p* < 0.001, **** *p* < 0.0001, and CTRL group compared to the PLN group; +++ *p* < 0.001, and AmLN and pAmLN groups compared to the PLN group.

**Figure 2:**
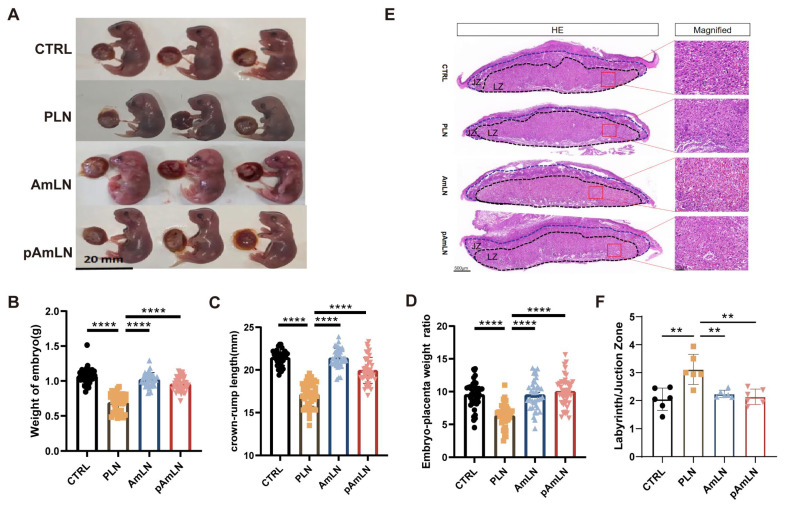
Pasteurized *Akkermansia muciniphila* improves placental and fetal development in PE mouse models. (**A**) Gross placental and fetal morphology across the experimental groups. (**B**) Fetal weight. (**C**) Fetal crown-rump length. (**D**) fetal/placental weight ratios. (**E**) Effects of pAKK and live AKK on placental morphology. Representative H&E-stained placental images are shown, with the labyrinth layer and junctional zone marked by dashed lines. Scale bars: 500 µm (**left**) and 50 µm (**right**). (**F**) Ratio of the labyrinth layer to the junctional zone in the placenta. The data are presented as the mean ± SEM (n = 6/group). ** *p* < 0.01, **** *p* < 0.0001, as determined using a one-way ANOVA following Bonferroni’s post hoc test. LZ, labyrinth zone; JZ, junctional zone.

Previous research has demonstrated that live AKK can mitigate PE-like symptoms in mice. Given the safety concerns associated with live bacteria, this study evaluated whether pAKK could provide similar benefits. As shown in Figure 1A, pregnant mice on day 8.5 were randomly assigned to four groups: control (CTRL), PE model (PLN), live AKK treatment (AmLN), and pasteurized AKK treatment (pAmLN). L-NAME exposure significantly increased the systolic blood pressure (SBP) and urine protein levels in the PLN group, confirming the successful establishment of the PE model (Figure 1B,C). Both pAKK and AKK pretreatments effectively prevented these increases (Figure 1B,C), indicating potential efficacy in reducing PE-associated hypertension and renal impairment. Fetal resorption rates (Figure 1E) were significantly improved by both treatments, demonstrating their potential for reducing fetal growth restriction. These improvements were further supported by an increased fetal weight (Figure 2B), crown-rump length (Figure 2C), and fetal/placental weight ratio (Figure 2D). In addition, the placental morphology (Figure 2E) and labyrinth/junctional zone ratios (Figure 2F) revealed that both pAKK and live AKK treatments significantly ameliorated L-NAME-induced placental abnormalities. These findings suggest that pAKK offers similar therapeutic benefits to live bacteria in alleviating PE symptoms, supporting its potential as a safer alternative for PE treatment.

### 3.2. Pasteurized Akkermansia muciniphila Mitigates Intestinal Barrier Damage in L-NAME-Induced PE Mice

**Figure 3:**
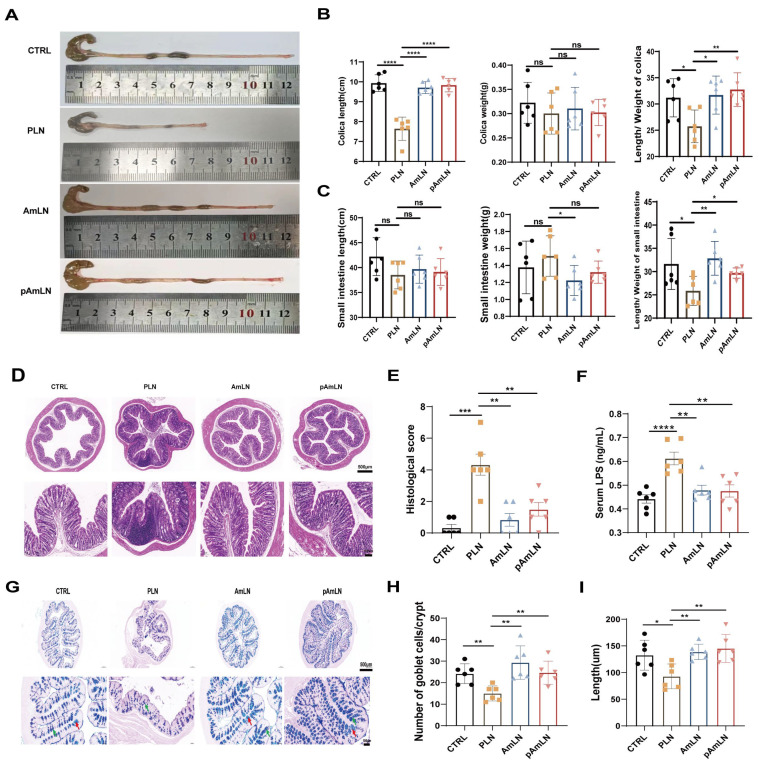
Pasteurized *Akkermansia muciniphila* improves intestinal barrier damage in PE mice stimulated by L-NAME. The mice were treated with PBS (CTRL), L-NAME (PLN), live AKK (AmLN), or pAKK(pAmLN) according to the animal experimental protocol described in the Methods section. (**A**) Digital images of colon tissues resected from the cecum to the rectum. (**B**) Average colon length (**left**), average colon weight (**middle**), and length/weight ratio of the colon (**right**) after treatment. (**C**) Average length (**left**), weight (**middle**), and length/weight ratio (**right**) of the small intestine after treatment. (**D**) Representative H&E-stained images of the proximal colon. The upper scale bar represents 500 µm, and the lower scale bar represents 100 µm. (**E**) Histological injury scores of the colon. (**F**) Portal plasma LPS levels (ng/mL). (**G**) Representative AB-PAS-stained images of the proximal colon. The upper scale bar represents 500 µm, and the lower scale bar represents 100 µm. Red arrows indicate acidic mucin and green arrows indicate mixed mucin. A total of 17–23 crypts (4–6 per colonic section) were randomly selected per animal to determine the number of goblet cells per crypt (**H**) as well as the depth of the crypt (**I**). The data are presented as the mean ± SEM (n = 6/group). * *p* < 0.05, ** *p* < 0.01, *** *p* < 0.001, **** *p* < 0.0001, ns, no significance, as determined using a one-way ANOVA following Bonferroni’s post hoc test.

**Figure 4:**
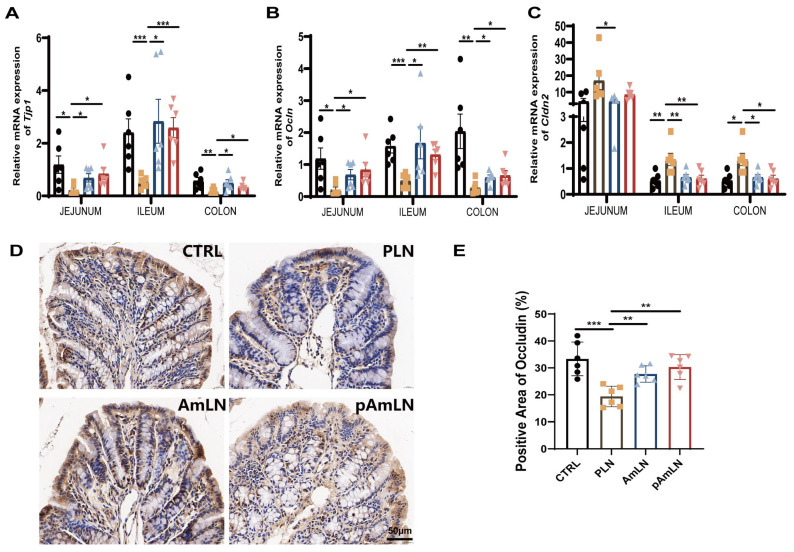
Pasteurized *Akkermansia muciniphila* enhances intestinal barrier integrity by regulating tight junction protein expression. (**A**–**C**) Relative mRNA expression of gut barrier function markers in the jejunum, ileum, and colon. Tight junction proteins: (**A**) *Tjp1*, (**B**) *Ocln*, and (**C**) *Cldn2*. (**D**) Representative image of occludin immunohistochemistry staining in colon tissues. Scale bar represents 50 µm. (**E**) Quantitative comparison of occludin expression levels. The data are presented as the mean ± SEM (n = 6/group). * *p* < 0.05, ** *p* < 0.01, *** *p* < 0.001, as determined using a one-way ANOVA following Bonferroni’s post hoc test.

Given the known association between gut microbiota imbalance and PE, we evaluated the effects of pAKK on intestinal barrier function in the L-NAME-induced PE mouse model. Previous studies have shown that live AKK improves intestinal barrier integrity and reduces gut permeability, thus alleviating PE symptoms. Here, we aimed to investigate whether pAKK provides similar benefits. Digital images of colon tissues (Figure 3A) revealed differences in the intestinal morphology among the different groups. The colon and small intestine length/weight ratios were significantly greater in both the pAmLN and AmLN groups (Figure 3B,C), indicating effective restoration of intestinal integrity. HE staining of the proximal colon (Figure 3D) revealed less tissue damage in the pAmLN and AmLN groups than in the PLN group, as confirmed by the lower histological injury scores (Figure 3E). The plasma LPS levels (Figure 3F) were significantly reduced in the pAmLN and AmLN groups, suggesting enhanced intestinal barrier function and reduced endotoxin leakage. AB-PAS staining (Figure 3G) revealed a notable increase in the acidic and mixed mucin distributions, indicating substantial recovery of the mucus layer. Both the number of goblet cells and the crypt depth (Figure 3H,I) were significantly greater in the pAmLN and AmLN groups than in the control group, confirming the restoration of the mucus layer. Furthermore, the pAKK treatment upregulated the expression of *Tjp1* and *Ocln* while downregulating *Cldn2* (Figure 4A–C). The IHC analysis further demonstrated reduced occludin protein levels in the PE model group (PLN), which were restored by the pAKK and live AKK treatments (Figure 4D,E). These results demonstrate that pAKK improves gut health and alleviates PE symptoms by enhancing intestinal barrier function. Future studies should focus on validating these results in clinical settings and further exploring the role of intestinal barrier function in pregnancy-related complications, particularly PE.

### 3.3. Pasteurized Akkermansia muciniphila Enhances Placental Angiogenesis and NO Synthesis in L-NAME-Induced PE Mice

**Figure 5:**
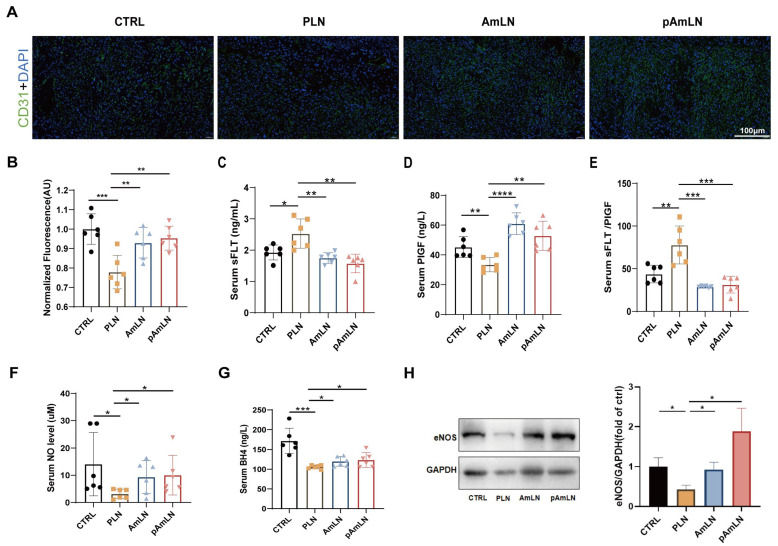
Pasteurized *Akkermansia muciniphila* improves placental angiogenesis and NO synthesis in PE mice stimulated by L-NAME. The mice were treated with PBS (CTRL), L-NAME (PLN), live AKK (AmLN), or pAKK(pAmLN) according to the animal experimental protocol described in the Methods section. (**A**,**B**) Immunofluorescence (IF) staining of CD31 in the placenta across the four groups. (**A**) Representative images are shown. (**B**) Quantification of IF intensity analyzed via ImageJ. Scale bar = 100 µm. (**C**) Serum sFlt-1 levels. (**D**) Serum PlGF levels. (**E**) sFlt-1/PlGF ratio. (**F**) Serum NO levels. (**G**) Serum tetrahydrobiopterin (BH4, a key cofactor of eNOS) levels. (**H**) Placental eNOS expression was detected by Western blotting. The data are presented as the mean ± SEM (n = 6/group). * *p* < 0.05, ** *p* < 0.01, *** *p* < 0.001, **** *p* < 0.0001, as determined using a one-way ANOVA followed by Bonferroni’s post hoc test.

To assess the therapeutic potential of pAKK in PE, this study examined its effects on placental and endothelial function in an L-NAME-induced PE mouse model. L-NAME, a nitric oxide synthase inhibitor, mimics the angiogenic and endothelial dysfunction observed in PE by reducing NO production. The IF staining revealed a significant increase in CD31 expression in both the pAmLN and AmLN groups, indicating enhanced placental angiogenesis (Figure 5A,B). These groups also presented significantly lower sFlt-1 levels, higher PlGF levels, and a reduced sFlt-1/PLGF ratio, reflecting improved placental function and angiogenesis (Figure 5C–E). The serum NO levels were markedly elevated in both treatment groups, indicating restored endothelial function (Figure 5F). Additionally, the BH4 levels were significantly increased, supporting enhanced endothelial nitric oxide synthase (eNOS) activity (Figure 5G), which was further confirmed by upregulated eNOS protein expression in the placenta (Figure 5H). These findings demonstrate that pAKK improves both placental angiogenesis and endothelial function, providing strong evidence for its potential application in treating PE. This study offers a novel strategy for managing PE by targeting the gut microbiota and endothelial function.

### 3.4. Pasteurized Akkermansia muciniphila Ameliorates Angiogenic Potential and NO Production in HUVECs Exposed to L-NAME In Vitro

**Figure 6:**
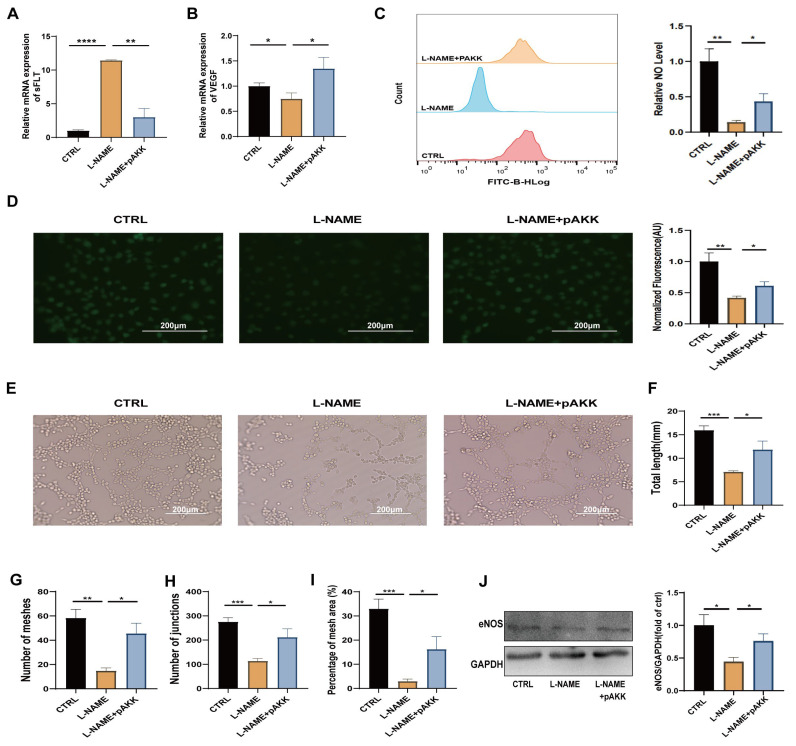
Pasteurized *Akkermansia muciniphila* ameliorates angiogenic potential and NO production in HUVECs exposed to L-NAME in vitro. Human umbilical vein endothelial cells (HUVECs) were incubated with pAKK for 24 h before L-NAME treatment (300 μM). (**A**,**B**) Gene expression levels of the antiangiogenic factor *sFlt-1* (**A**) and the proangiogenic factor *VEGFA* (**B**) in different HUVEC groups. (**C**,**D**) Intracellular NO levels in HUVECs were measured via the DAF-FM DA probe. (**C**) Flow cytometry analysis of intracellular NO levels in HUVECs. (**D**) Fluorescence imaging of intracellular NO levels in HUVECs, with representative images captured at 200× magnification (left panel) and quantification of fluorescence intensity (right panel). (**E**–**I**) pAKK enhances the angiogenic capacity of HUVECs stimulated with L-NAME. (**E**) Tube formation of HUVECs treated with CTRL (control), L-NAME, or L-NAME + pAKK. The total tubule length (**F**), number of meshes (**G**), number of junctions (**H**), and percentage of mesh area (**I**) were measured via quantitative analysis at 6 h postinduction. (**J**) eNOS expression in different HUVEC groups, with eNOS and GAPDH levels detected by Western blotting. The data are presented as the mean ± SEM (n = 4). * *p* < 0.05, ** *p* < 0.01, *** *p* < 0.001, **** *p* < 0.0001, as determined using a one-way ANOVA following Bonfferoni’s post hoc test.

To explore the effects of pAKK on the endothelial cells, HUVECs were used as an in vitro model. As expected, the L-NAME treatment significantly impaired endothelial function by reducing NO production and angiogenic capacity. Pretreatment with pAKK effectively reversed these impairments. Specifically, pAKK decreased the expression of the antiangiogenic factor sFlt-1 while increasing the expression of the proangiogenic factor vascular endothelial growth factor (VEGF), indicating enhanced angiogenesis (Figure 6A,B). Flow cytometry and fluorescence imaging via the DAF-FM DA probe confirmed that pAKK significantly elevated the intracellular NO levels in HUVECs, further confirming its positive impact on endothelial function (Figure 6C,D). In the tube formation assays, pAKK significantly improved the angiogenic metrics, including the total tubule length, the number of meshes, junctions, and the mesh area percentage (Figure 6E–I). Additionally, Western blot analysis revealed a marked increase in endothelial nitric oxide synthase (eNOS) expression in the pAKK-treated group compared with the L-NAME group (Figure 6J), supporting the role of pAKK in enhancing NO synthesis. These findings demonstrate that pAKK mitigates L-NAME-induced endothelial dysfunction by enhancing angiogenesis and NO production. These findings support its potential application in treating endothelial dysfunction in PE and provide insights for the development of new therapeutic strategies.

### 3.5. Pasteurized Akkermansia muciniphila Reverses Fecal Metabolomics Disorders in PE Mice

**Figure 7:**
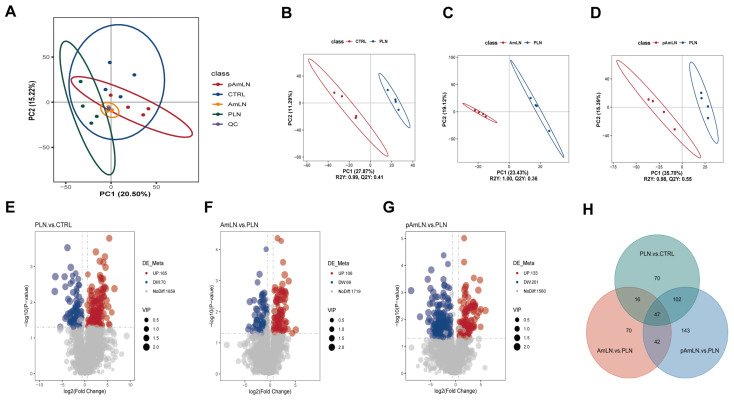
Pasteurized *Akkermansia muciniphila* reverses fecal metabolomics disorders in PE mice. (**A**) PCA results: The x-axis represents the first principal component (PC1), and the y-axis represents the second principal component (PC2). The ellipses indicate the 95% confidence intervals. Each dot represents a sample, with different colors corresponding to different groups. (**B**–**D**) PLS-DA results: the *x*-axis represents the first principal component, and the *y*-axis represents the second principal component. The numbers in parentheses indicate the percentage of total variance explained by the corresponding principal component. (**E**–**G**) Volcano plot analysis: the *x*-axis represents the log2(fold change), and the *y*-axis represents the −log10(*p*-value). Each point represents a metabolite. Significantly upregulated metabolites are shown as red dots and significantly downregulated metabolites are shown as blue dots. The size of each dot indicates the VIP value. (**H**) Venn diagram: overlapping and unique differentially abundant metabolites among the PLN vs. CTRL, AmLN vs. PLN, and pAmLN vs. PLN comparisons.

To evaluate the therapeutic potential of pAKK in PE, untargeted fecal metabolomics was performed on mice from the CTRL, PLN, AmLN, and pAmLN groups. The quality control (QC) samples showed tight clustering in the PCA, confirming the reliability of the data (Figure 7A). PLS-DA further revealed significant metabolic disturbances in the PLN group compared with those in the CTRL group, which were partially reversed by both pAKK and AKK treatments (Figure 7B–D). These findings suggest that pAKK may alleviate PE symptoms by modulating the metabolic balance, similar to live AKK. The volcano plot analysis revealed that 165 metabolites were upregulated and 70 were downregulated in the PLN group compared with the CTRL group, whereas 133 metabolites were upregulated and 201 were downregulated in the pAmLN group, suggesting that the therapeutic effects of pAKK may involve the modulation of these metabolites (Figure 7E–G). The Venn diagram analysis compared overlapping and unique differentially abundant metabolites among the PLN vs. CTRL, AmLN vs. PLN, and pAmLN vs. PLN groups. The analysis revealed 42 common differentially abundant metabolites across the three groups (Supplementary Table S2), with 26 metabolites upregulated in the PE model group and downregulated in the pAKK and AKK treatment groups and 21 metabolites downregulated in the PE model group and upregulated in the treatment groups (Figure 7H). These results highlight the potential of pAKK to correct PE-related metabolic disturbances through the regulation of specific metabolites, similar to live AKK.

### 3.6. Metabolic Pathway Enrichment Analysis of Differential Metabolites

**Figure 8:**
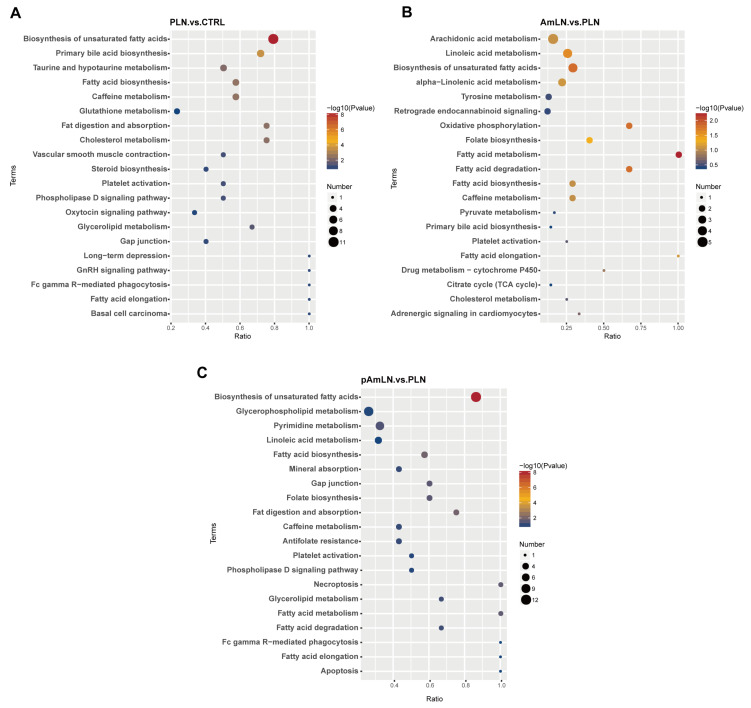
Metabolic pathway enrichment analysis of differential metabolites. (**A**–**C**) The *x*-axis represents the enrichment factor, with a higher value indicating a greater ratio of differentially abundant metabolites annotated to the pathway. The color of the dots represents the *p*-value from the hypergeometric test, with smaller values indicating greater statistical significance. The size of the dots indicates the number of differentially abundant metabolites annotated to each pathway.

KEGG enrichment analysis was conducted to explore the metabolic pathways involved in PE improvement by pAKK and AKK treatments, comparing the PLN vs. CTRL, AmLN vs. PLN, and pAmLN vs. PLN groups. In the PLN vs. CTRL and AmLN vs. PLN comparisons, 16 overlapping pathways were identified, including fatty acid metabolism, biosynthesis of unsaturated fatty acids, linoleic acid metabolism, folate biosynthesis, and adrenergic signaling in cardiomyocytes (Figure 8A,B, Supplementary Table S3). These pathways suggest that live AKK alleviates PE-induced metabolic disturbances through broad metabolic regulation. Similarly, the PLN vs. CTRL and pAmLN vs. PLN comparisons revealed 44 overlapping pathways, including the biosynthesis of unsaturated fatty acids, fatty acid metabolism, folate biosynthesis, pyrimidine metabolism, primary bile acid biosynthesis, and ferroptosis (Figure 8A,C, Supplementary Table S4). These findings indicate that pAKK improves PE symptoms through multipathway regulation, similar to live AKK. Additionally, 13 pathways were consistently enriched across the PLN vs. CTRL, AmLN vs. PLN, and pAmLN vs. PLN comparisons, involving key processes such as unsaturated fatty acid biosynthesis, fatty acid degradation, folate biosynthesis, and adrenergic signaling in cardiomyocytes (Figure 8A–C, Supplementary Table S5). These common pathways may provide important insights into PE pathogenesis and the therapeutic mechanisms of AKK and pAKK. In summary, both pAKK and live AKK modulate critical metabolic pathways, including unsaturated fatty acid biosynthesis, folate metabolism, and linoleic acid metabolism, to alleviate PE symptoms. These findings support the potential of pAKK as a promising adjunctive therapy for PE and highlight the expanding role of microbiome-based interventions in targeting metabolic dysregulation associated with the condition.

## 4. Discussion

Our results revealed that pAKK significantly reduced the systolic blood pressure, urinary protein levels, and fetal resorption rates and alleviated fetal growth restriction in PE mice, which aligns with the findings of previous studies in which live AKK was used [19]. Interestingly, pasteurization of AKK has been shown to enhance its effectiveness in metabolic disorder models [8], and human trials have validated this in the context of obesity and related diseases [12]. Compared with live bacteria, pasteurization reduces the risks associated with gut dysbiosis and infection, making pAKK a safer therapeutic option. Moreover, pAKK significantly improved L-NAME-induced gut barrier dysfunction in PE mice. These improvements in gut barrier function align with the findings from studies on metabolic syndrome and inflammatory bowel disease, further supporting the potential of pAKK as a safe and effective adjunctive therapeutic alternative [20,21].

The placenta plays a pivotal role in the development of PE, particularly through its impact on vascular dysfunction. Placenta-derived factors disrupt maternal endothelial function, leading to an imbalance between proangiogenic and antiangiogenic factors, impaired angiogenesis, and abnormal vascular tone [22]. Notably, reduced levels of NO, a key regulator of endothelial function primarily produced by the placenta during pregnancy, are closely associated with the onset of PE [23,24]. Emerging evidence suggests that the gut microbiota may influence NO production and, consequently, endothelial function [25,26]. For example, inulin-type fructans have been shown to improve endothelial dysfunction by increasing AKK abundance and modulating the eNOS-NO pathway [27]. In our study, we utilized L-NAME, a nitric oxide synthase inhibitor, to induce a PE-like model characterized by endothelial dysfunction [28]. This model underscores the crucial role of the imbalance between proangiogenic (PlGF) and antiangiogenic (sFlt-1) factors in PE pathogenesis. Elevated sFlt-1 levels impair placental angiogenesis and exacerbate disease progression in PE patients [29]. The sFlt-1/PlGF ratio is increasingly recognized as a valuable diagnostic and prognostic marker for PE [30], and the Food and Drug Administration (FDA) recently approved a blood test for this ratio as a biochemical predictor of the condition [31]. Our findings demonstrated that pAKK significantly reduced the sFlt-1/PlGF ratio in PE mice, thereby promoting placental angiogenesis. In addition, pAKK improved endothelial dysfunction by increasing the serum NO levels and increasing placental eNOS expression. This effect was further supported by the ability of pAKK to restore the levels of BH4, a critical cofactor for eNOS, whose deficiency shifts eNOS from producing NO to generating superoxide, thereby worsening endothelial dysfunction [32]. pAKK effectively reversed the L-NAME-induced suppression of BH4 expression in both in vivo and in vitro models, contributing to restored NO biosynthesis. Furthermore, NO produced by uterine endothelial cells is vital for embryonic development, implantation, trophoblast invasion, and placental vascular development [33]. Our in vitro angiogenesis assays using HUVECs further confirmed that pAKK enhances endothelial function by restoring NO biosynthesis. These results align with recent studies, emphasizing that regulating NO levels is crucial for alleviating PE-related vascular dysfunction and that improving endothelial function can effectively mitigate PE symptoms [28,34].

The role of the gut microbiota in various health and disease states is increasingly recognized, but our study focused on metabolomics rather than 16S rRNA sequencing. This decision was supported by previous studies showing that neither live AKK nor pAKK treatment significantly altered the gut microbiota composition in mice [35] or humans [12]. A systematic review also revealed that most human probiotic studies did not show significant changes in the microbiota composition [36]. Therefore, we hypothesize that the therapeutic effects of live or pasteurized AKK primarily arise from alterations in microbiota metabolic activity rather than changes in composition [20].

In this study, the metabolomic analysis revealed significant metabolic disturbances in the PE model, which were partially reversed by both AKK and pAKK treatments. We identified 42 differentially abundant metabolites that followed similar trends in both treatment groups, closely correlating with improved PE symptoms. Previous studies have shown that lipid and amino acid metabolites are significantly altered in the serum of PE patients and that their restoration is essential for alleviating PE [37,38]. Animal studies further suggest that targeting gut microbiota-derived metabolites could help improve PE outcomes [6,9,39]. For example, sodium butyrate supplementation in rats has been shown to alleviate PE-like symptoms by improving the gut microbiota and increasing short-chain fatty acid (SCFA) production [39]. Additionally, metabolites such as trimethylamine N-oxide (TMAO) have emerged as potential therapeutic targets for PE [6].

Our KEGG pathway enrichment analysis identified several key metabolic pathways through which pAKK may alleviate PE symptoms. Among these, the biosynthesis of unsaturated fatty acids is crucial for maintaining cell membrane fluidity and mitigating oxidative stress, both of which play significant roles in PE pathogenesis [40,41]. Dysregulation of this pathway is linked to increased oxidative damage in PE, which is consistent with the prior findings that maternal imbalances in unsaturated fatty acids may increase a PE risk. This underscores the importance of a balanced intake of ω-3 and ω-6 polyunsaturated fatty acids (PUFAs) during pregnancy [42]. A meta-analysis further revealed that ω-3 PUFA supplementation during pregnancy can prevent PE, particularly when it is initiated early [43]. Furthermore, PUFAs have been recognized as a cornerstone therapy for managing hypertriglyceridemia, a condition closely associated with an increased risk of preeclampsia and vascular dysfunction [44]. However, while the potential benefits of PUFA supplementation during pregnancy have been suggested, their use is not yet widely studied, and current evidence does not support its routine recommendation during pregnancy [45]. Further research is warranted to explore the safety and efficacy of PUFA supplementation as an adjunctive therapy for PE management. Additionally, linoleic acid metabolism and folate biosynthesis pathways were identified as critical in PE pathogenesis. Dysregulation of linoleic acid metabolism may worsen hypertension and endothelial dysfunction [46,47], whereas proper regulation of folate biosynthesis is vital for preventing pregnancy complications [48,49]. Studies have shown that the folate biosynthesis pathway is more enriched in the gut microbiota of healthy pregnant women than in PE patients [50], suggesting that pAKK, similar to live AKK, may reverse PE-associated metabolic disturbances by regulating folate synthesis. This study demonstrated that pAKK can restore metabolic homeostasis in a PE model, with effects comparable to those of live bacteria. These findings contribute to the expanding body of research on PE-related gut metabolomics and provide novel insights into the therapeutic potential of gut-derived metabolites for PE treatment. Prior studies have established a close link between the gut microbiota and pregnancy metabolic syndrome [51], whereas research on Chinese PE patients has highlighted the therapeutic role of gut metabolites in managing PE [52], supporting our findings.

Despite these promising results, this study has certain limitations. It was conducted in a mouse model, which, while valuable, may not fully replicate the complexity of human PE. Further investigations, including clinical trials, are needed to validate these findings in human populations. Additionally, the precise molecular mechanisms through which pAKK exerts its effects remain unclear. Future research should focus on elucidating these mechanisms and investigating the long-term impact of pAKK treatment on maternal and neonatal health.

In conclusion, this study is the first to demonstrate that pAKK significantly improves endothelial dysfunction and gut barrier integrity in a PE mouse model via modulation of the eNOS/NO pathway and gut microbiota-derived metabolites. Compared with live AKK, pAKK provides a safer, equally effective alternative. These findings highlight the potential of pAKK and gut metabolites in preventing and treating PE. Future clinical trials targeting the eNOS/NO pathway and gut microbiota metabolites will be crucial for advancing PE management strategies. This study provides a solid scientific foundation for the development of gut microbiota-based therapies for PE and broadens the scope for postbiotic product applications in clinical practice.

## Data Availability

Mouse fecal metabolomics raw data from the CTRL, PLN, AmLN, and pAmLN groups have been deposited in Mendeley Data and can be accessed at Peng, Linyu (2024), “Mouse fecal metabolomics raw data from CTRL, PLN, AmLN, and pAmLN”, Mendeley Data, V1, doi: 10.17632/498c4cbvnt.1 [18]. Other data that support the findings of this study are available from the corresponding author upon reasonable request.

## Supplementary Materials

The following supporting information can be downloaded at: https://www.mdpi.com/article/10.3390/microorganisms12122483/s1, Table S1: The primers for quantitative real-time PCR in this study; Table S2: 42 common differential metabolites across PLN vs. CTRL, AmLN vs. PLN, and pAmLN vs. PLN; Table S3: Overlapping metabolic pathways identified in PLN vs. CTRL and AmLN vs. PLN comparisons; Table S4: Overlapping metabolic pathways identified in PLN vs. CTRL and pAmLN vs. PLN comparisons; Table S5: Overlapping metabolic pathways identified in PLN vs. CTRL, AmLN vs. PLN, and pAmLN vs. PLN comparisons.

## Abbreviations

PE	Preeclampsia
AKK	*Akkermansia muciniphila*
Pakk	Pasteurized *Akkermansia muciniphila*
LC-MS/MS	Liquid chromatography-tandem mass spectrometry
eNOS	Endothelial nitric oxide synthase
NO	Nitric oxide
HUVECs	Human umbilical vein endothelial cells
EFSA	European Food Safety Authority
TLR2	Toll-like receptor 2
LPS	Lipopolysaccharide
NIH	National institutes of health
HE	Hematoxylin and eosin
IF	Immunofluorescence
AB-PAS	Alcian blue periodic acid Schiff
sFlt-1	Serum soluble Fms-like tyrosine kinase-1
PlGF	Placental growth factor
BH4	Tetrahydrobiopterin
qRT-PCR	Quantitative real-time PCR
ECL	Enhanced chemiluminescence
PCA	Principal component analysis
PLS-DA	Partial least squares discriminant analysis
QC	Quality control
SCFA	Short-chain fatty acid
TMAO	Trimethylamine N-oxide
PUFAs	Polyunsaturated fatty acids
